# *NDUFA4* Mutations Underlie Dysfunction of a Cytochrome *c* Oxidase Subunit Linked to Human Neurological Disease

**DOI:** 10.1016/j.celrep.2013.05.005

**Published:** 2013-06-27

**Authors:** Robert D.S. Pitceathly, Shamima Rahman, Yehani Wedatilake, James M. Polke, Sebahattin Cirak, A. Reghan Foley, Anna Sailer, Matthew E. Hurles, Jim Stalker, Iain Hargreaves, Cathy E. Woodward, Mary G. Sweeney, Francesco Muntoni, Henry Houlden, UK10K Consortium, Jan-Willem Taanman, Michael G. Hanna

**Affiliations:** 1 MRC Centre for Neuromuscular Diseases, UCL Institute of Neurology and National Hospital for Neurology and Neurosurgery, Queen Square, London WC1N 3BG, UK; 2 Mitochondrial Research Group, Clinical and Molecular Genetics Unit, UCL Institute of Child Health, London WC1N 1EH, UK; 3 Neurogenetics Unit, National Hospital for Neurology and Neurosurgery, Queen Square, London WC1N 3BG, UK; 4 Neurometabolic Unit, National Hospital for Neurology and Neurosurgery, Queen Square, London WC1N 3BG, UK; 5 Dubowitz Neuromuscular Centre, UCL Institute of Child Health and Great Ormond Street Hospital for Children NHS Foundation Trust, London WC1N 1EH, UK; 6 Department of Molecular Neuroscience, UCL Institute of Neurology, Queen Square, London WC1N 3BG, UK; 7 The Wellcome Trust Sanger Institute, Wellcome Trust Genome Campus, Hinxton, Cambridge CB10 1SA, UK; 8 Department of Clinical Neuroscience, UCL Institute of Neurology, London NW3 2PF, UK

## Abstract

The molecular basis of cytochrome *c* oxidase (COX, complex IV) deficiency remains genetically undetermined in many cases. Homozygosity mapping and whole-exome sequencing were performed in a consanguineous pedigree with isolated COX deficiency linked to a Leigh syndrome neurological phenotype. Unexpectedly, affected individuals harbored homozygous splice donor site mutations in *NDUFA4*, a gene previously assigned to encode a mitochondrial respiratory chain complex I (NADH:ubiquinone oxidoreductase) subunit. Western blot analysis of denaturing gels and immunocytochemistry revealed undetectable steady-state NDUFA4 protein levels, indicating that the mutation causes a loss-of-function effect in the homozygous state. Analysis of one- and two-dimensional blue-native polyacrylamide gels confirmed an interaction between NDUFA4 and the COX enzyme complex in control muscle, whereas the COX enzyme complex without NDUFA4 was detectable with no abnormal subassemblies in patient muscle. These observations support recent work in cell lines suggesting that NDUFA4 is an additional COX subunit and demonstrate that *NDUFA4* mutations cause human disease. Our findings support reassignment of the NDUFA4 protein to complex IV and suggest that patients with unexplained COX deficiency should be screened for *NDUFA4* mutations.

## Highlights

• Mutations in *NDUFA4*, assigned to encode a complex I subunit, cause human COX deficiency
• Confirmed interaction between NDUFA4 and the COX holoenzyme in control muscle
• The COX holoenzyme without NDUFA4 is detectable with no abnormal subassemblies in patient muscle
• NDUFA4 is essential for complex IV activity, but is not required for assembly of the COX holoenzyme

## Graphical Abstract

**Figure:**
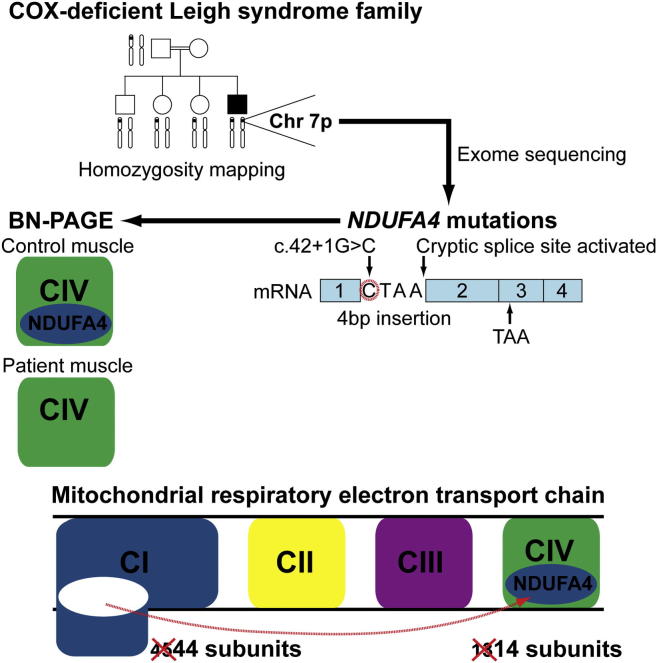

## Teaser

Isolated cytochrome *c* oxidase (COX) deficiency is a frequent finding in human mitochondrial disease. Mutations in nuclear-encoded structural subunits are extremely rare, and, in many cases, the molecular basis remains undetermined. Recent evidence in cell lines has suggested that *NDUFA4*, previously assigned to encode a complex I subunit, actually encodes a structural component of COX. Hanna and colleagues now demonstrate that *NDUFA4* mutations cause human COX deficiency, thus confirming NDUFA4 as a COX subunit that is essential for the enzyme’s activity.

## Introduction

Cytochrome *c* oxidase (COX, complex IV) is the terminal enzyme complex of the mitochondrial respiratory chain and plays a vital role in cellular energy transformation. Mammalian COX is reported to have a crystal structure comprising 13 polypeptide subunits (Tsukihara et al., 1996). The three largest subunits are encoded by the mitochondrial DNA (mtDNA) and form the catalytic core of the enzyme. The remaining ten subunits are nuclear-encoded and are thought to have a function in assembly/stability and dimerization of the enzyme, and regulation of the enzyme’s catalytic activity (Taanman, 1997a). Mutations in the structural subunits are extremely rare (Hanna et al., 1998; Rahman et al., 1999) with only three nuclear-encoded COX subunits linked to human disease (Massa et al., 2008; Shteyer et al., 2009; Indrieri et al., 2012). To date, most cases of isolated COX deficiency are caused by mutations in nuclear-encoded proteins required for COX translation, maturation, or assembly (Soto et al., 2012). Furthermore, these reported nuclear gene mutations are typically associated with severe neonatal or childhood-onset presentations and an early fatal outcome. However, many cases of COX deficiency remain undefined at the molecular level.

We investigated the genetic basis of neurological disease in a large consanguineous Pakistani family in whom four affected relatives had isolated COX deficiency. The natural history was of an initial presentation with congenital lactic acidosis and subsequent evolution into a Leigh syndrome (Mendelian Inheritance in Man [MIM] 256000) neurological phenotype with bulbar dysfunction, dystonia, ataxia, spasticity, and intermittent encephalopathy. Whole-mtDNA sequencing was normal, and genetic analysis of nuclear genes known to cause isolated COX deficiency did not reveal any pathogenic mutations.

## Results

### Genetic Investigations

#### Homozygosity Mapping and Whole-Exome Sequencing Bioinformatic Analysis

**Figure 1:**
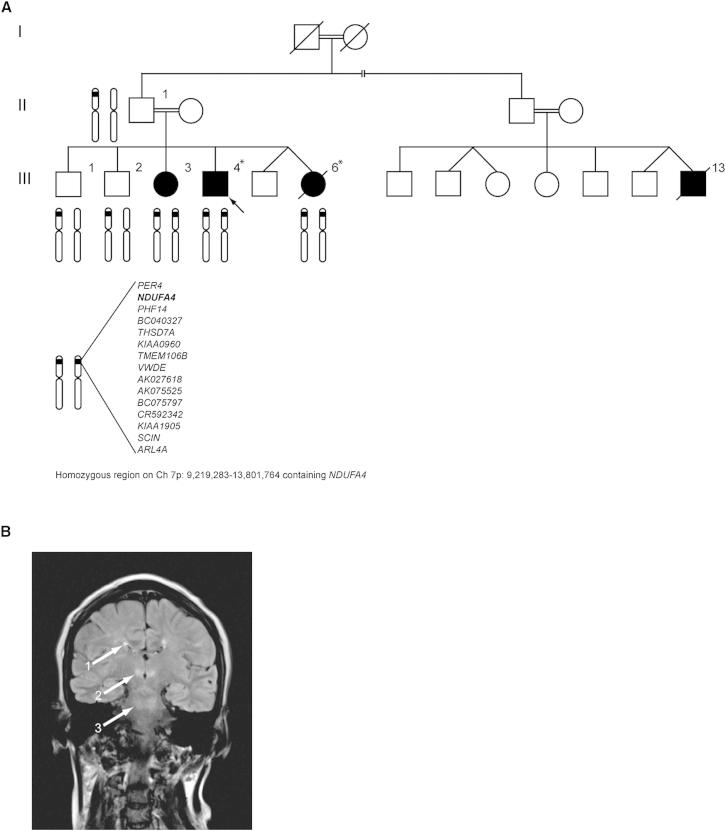
Pedigree of Family Harboring the c.42+1G → C Mutation in *NDUFA4* and Brain MRI Characteristic of Leigh Syndrome (A) Patient-reported pedigree of consanguineous family harboring the c.42+1G → C mutation in *NDUFA4* is shown. Arrow indicates proband. Members of the family whose genotype data were used in homozygosity mapping analyses are illustrated by chromosome 7 schematic. Those whose exomes were sequenced are marked with an asterisk (^∗^). Filled symbols indicate affected individuals. Square symbols indicate male gender. Round symbols indicate female gender. Symbols with diagonal strikethrough indicate deceased. Broken line indicates the possibility that III-13 is a more distant cousin. (B) MR T2-weighted coronal image of brain (III-3), characteristic of Leigh syndrome, demonstrating multiple scattered T2 hyperintensities in deep white matter of both cerebral hemispheres (arrow, 1), medial thalami (arrow, 2), and at the level of the superior cerebellar peduncles (arrow, 3). Hyperintensities were also evident in the corticospinal tracts of the pons, right middle cerebellar peduncles, cerebellar hemispheres, and descending tracts at the level of the medulla oblongata (data not shown). Similar appearances were evident on the brain MRI of the other affected patients. See also Table S1.

**Figure 2:**
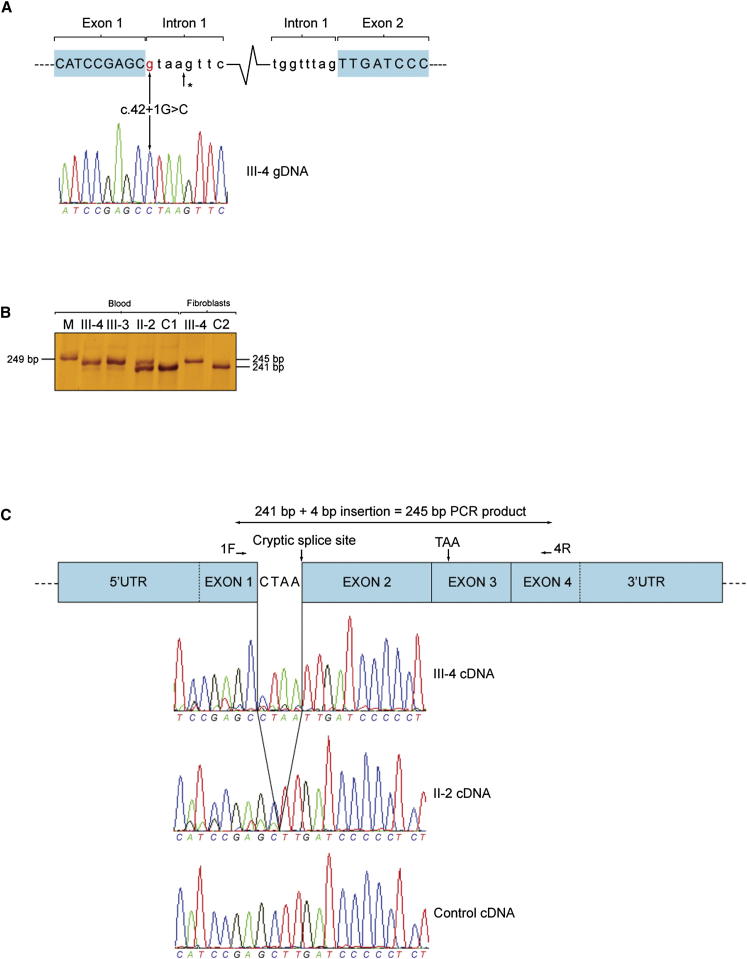
Activated Cryptic Splice Site Downstream to the c.42+1G → C Mutation Causes a Frameshift and Introduces a Premature Stop Codon in mRNA (A) Schematic demonstrating location of c.42+1G → C mutation within *NDUFA4* intron 1, and homozygous mutation in forward genomic DNA (gDNA) sequencing of an affected subject. Position of cryptic splice site activated adjacent to the next GT dinucleotide 4 bp downstream of c.42+1G → C is marked with an asterisk (^∗^). (B) Silver-stained polyacrylamide gel of amplified complementary DNA (cDNA) extracted from blood and cultured skin fibroblasts alongside a 249 bp molecular weight marker (M). In blood, III-3 and III-4 (affected siblings) have a strongly stained (mutant) band at 245 bp and a faintly stained (wild-type) band at 241 bp, whereas II-2 (unaffected father) has a strongly stained band at 241 bp and a faintly stained band at 245 bp. In cultured skin fibroblasts, III-4 only has a band at 245 bp. The control blood (C1) and cultured skin fibroblasts (C2) only have a band at 241 bp. (C) Schematic depicting the mutant *NDUFA4* transcript retaining 4 bp of intron 1, with sequencing electropherograms below (reverse sequencing shown). Close inspection of the mixed electropherogram 5′ of exon 2 in III-4 confirmed that wild-type sequence was present at low levels (also seen in III-3, data not shown). Mutant sequence can also be seen in II-2. Abbreviations: 1F and 4R, exonic primer binding sites; TAA, premature stop codon. See also Table S4.

To identify areas of shared homozygosity among affected relatives, we genotyped six family members (three affected and three unaffected, Figure 1A). Two large regions of shared homozygosity mapped to chromosome 7p (nucleotides 9,219,283–13,801,764, containing 15 protein-coding genes; and nucleotides 19,034,191–29,250,335, containing 92 protein-coding genes, Table S1). As no candidate genes for COX deficiency were present in either region, it was initially concluded that a small area of homozygosity had been overlooked. We therefore undertook whole-exome sequencing in two affected family members (III-4 and III-6). Our filtering pathway (Table 1) searched for novel (not reported to dbSNP132 and/or 1000 Genomes, the remaining UK10K rare disease cohort [823 exomes at the time of the analysis], or the NHLBI Exome Sequencing Project [ESP] database), homozygous (in view of parental consanguinity), functional (nonsynonymous coding and/or loss-of-function), single-nucleotide variants (SNVs) and/or coding insertions/deletions (indels) shared by both affected siblings. We initially searched genes predicted to play a role in COX biogenesis. However, using this strategy no candidate genes were identified across the entire exome. We subsequently relaxed our filtering strategy to include all known nuclear-encoded mitochondrial genes (Pagliarini et al., 2008) and identified a homozygous splice donor site mutation (c.42+1G → C, NM_002489.3) in *NDUFA4*, a gene within one of our previously identified regions of homozygosity (see Tables S2 and S3 for coverage of the linkage loci in III-4 and III-6). Sanger sequencing demonstrated that all four affected individuals were homozygous for the mutation, whereas three unaffected family members (II-2, III-1, and III-2) were heterozygous (Figure 2A). A further 20 probands with isolated COX deficiency were screened for mutations in *NDUFA4*, but no further variants were identified.

#### Transcriptional Analysis of c.42+1G → C Mutation

To study the effect the c.42+1G → C mutation had on mRNA splicing, we amplified *NDUFA4* complementary DNA (cDNA) fragments, generated from reverse transcription of mRNA extracted from whole blood and cultured skin fibroblasts, and resolved the PCR products on a denaturing 7% polyacrylamide gel followed by silver staining. This revealed two fragments in blood: (1) a band measuring 241 bp, corresponding to the wild-type transcript, which was present in all samples but at much lower levels in the affected subjects (III-3 and III-4) compared with an unaffected carrier (II-2) and the control; and (2) a band at 245 bp that was absent in the control sample but present at high levels in the affected subjects with lower levels detectable in the unaffected carrier. Cultured skin fibroblasts demonstrated a 245 bp fragment in the patient studied (III-4), with no evidence of wild-type transcript (Figure 2B). Low-level wild-type cDNA sequence was observed in the sequencing electropherograms of both affected individuals (Figure 2C, data for III-3 not shown). These data indicate that the c.42+1G → C mutation does not appear to completely abolish correct splicing of *NDUFA4* exon 1 to exon 2 in blood. Sanger sequencing of the PCR products revealed that the first 4 bp of intron 1 are retained following activation of a cryptic splice site 4 bp downstream of the c.42+1G → C mutation (Figures 2A and 2C). This frameshift is predicted to introduce a premature stop codon (TAA) 105 bp downstream in exon 3. If translated, the resultant protein would consist of 14 in-frame amino acids followed by 35 out-of-frame amino acids (compared to the 81 amino acid wild-type protein).

### Western Blot of Denaturing Polyacrylamide Gels and Immunocytochemistry

**Figure 3:**
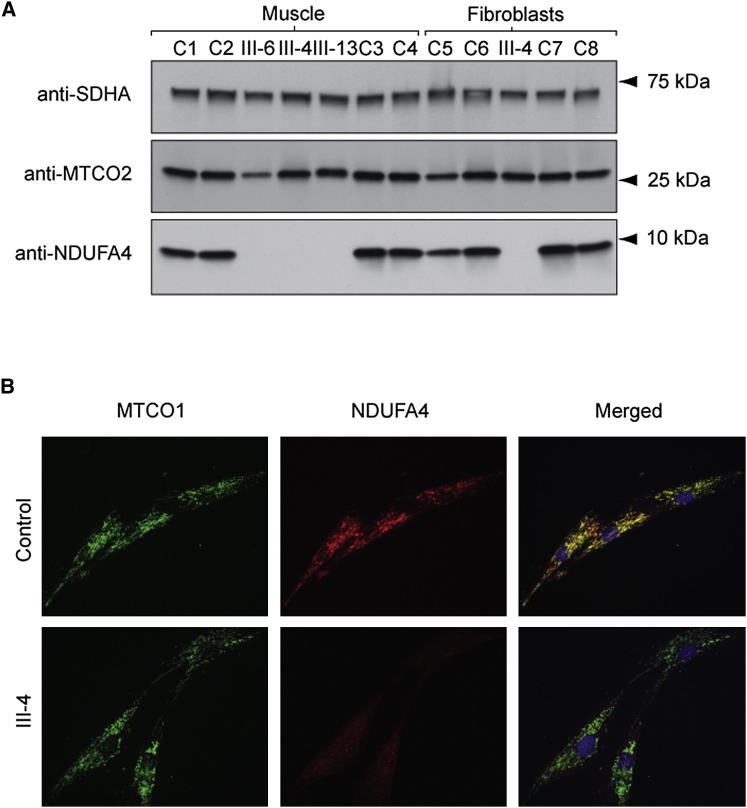
Steady-State NDUFA4 Protein Levels Are Undetectable in Muscle Tissue and Cultured Skin Fibroblasts of Patients Harboring Homozygous c.42+1G → C Mutations (A) Western blot analysis of an SDS polyacrylamide gel loaded with 10 μg of mitochondrial protein pellets extracted from muscle tissue and cultured skin fibroblasts of the affected subjects (III-4, III-6, III-13 muscle tissue, III-4 cultured skin fibroblasts) and controls (C1–8). Blots were developed with antibodies directed against SDHA, MTCO2, and NDUFA4. The antibody to complex II subunit SDHA was used to confirm equal protein loading. (B) Micrographs of control and affected subject (III-4) fibroblasts immunocytochemically stained for NDUFA4 (red fluorescence) and MTCO1 (green fluorescence) and counterstained with the DNA fluorochrome 4’,6-diamidino-2-phenylindole (blue fluorescence). Note that NDUFA4 is absent from patient cells, whereas MTCO1 levels are comparable to the control cells.

NDUFA4 protein was undetectable in all affected subjects examined (muscle tissue of III-4, III-6, and III-13, cultured skin fibroblasts of III-4). Steady-state level of the mitochondrially encoded COX subunit MTCO2 was markedly reduced in the muscle tissue of one patient compared with the mean MTCO2 value of four controls (MTCO2 level in III-6 56% of controls). The steady-state MTCO2 subunit levels in III-6 and III-13 were comparable to the control muscle tissue samples (Figure 3A). Immunocytochemistry also showed absent NDUFA4 protein in cultured skin fibroblasts (III-4) with normal mitochondrially encoded COX subunit MTCO1 (Figure 3B). MitoTracker staining confirmed colocalization of NDUFA4 in control fibroblasts (data not shown).

### In-Gel Activity and Western Blot Analysis of One- and Two-Dimensional Blue-Native Polyacrylamide Gels

**Figure 4:**
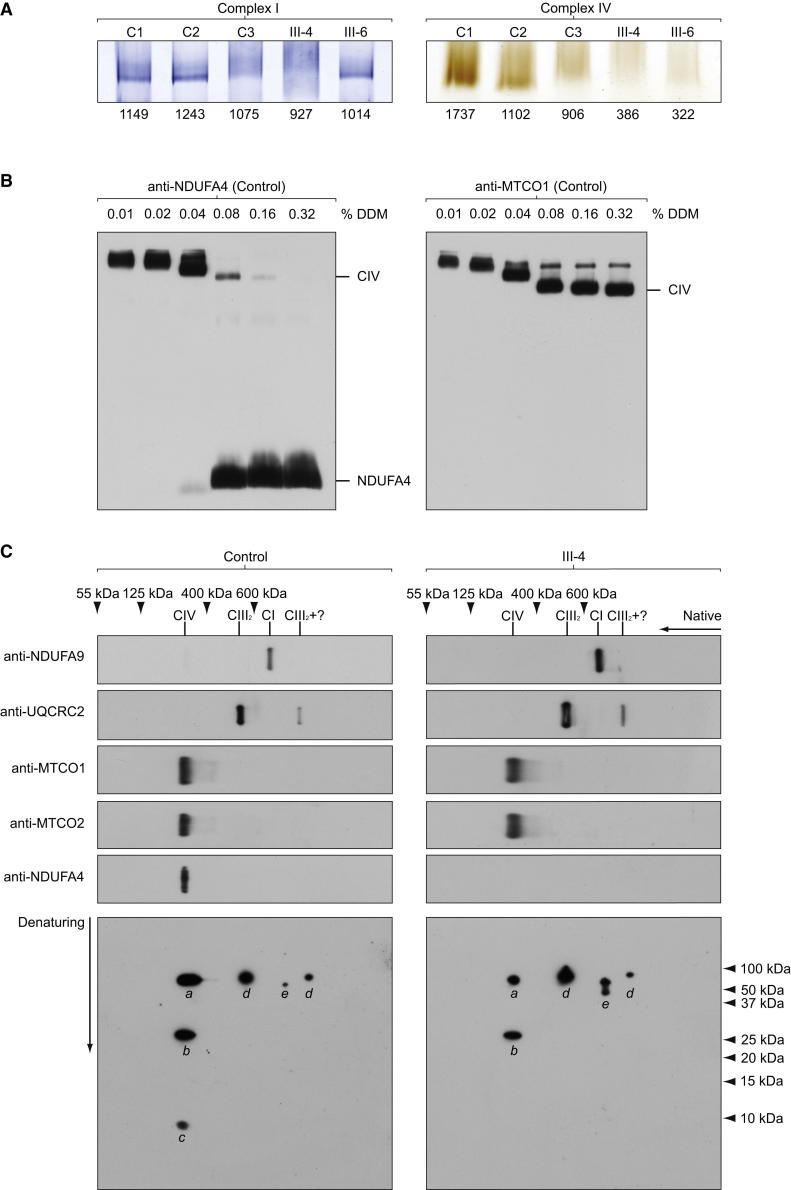
NDUFA4 Subunit and COX Enzyme Complex Interact in Control Muscle, whereas COX Enzyme Complex without NDUFA4 Subunit Is Detectable with No Abnormal Subassemblies in Patient Muscle (A) In-gel activity of complex I and IV in 3%–12% polyacrylamide blue-native gels loaded with 70 μg of mitochondrial enriched protein pellets extracted from control (C1–3) and patient (III-4 and III-6) muscle tissue. Complex I activity is comparable among the control and affected subjects. However, significant reduction in complex IV activity is demonstrated in patient muscle tissue. Smearing of complex I can be seen in C3 and III-4 due to high amount of protein loaded. The measured staining intensity is indicated below each lane. (B) Western blot analysis of blue-native polyacrylamide gels loaded with 10 μg of mitochondrial protein pellets extracted from control muscle tissue. The following concentrations of n-dodecyl β-D-maltoside (DDM) were used to extract mitochondrial membrane proteins: 0.01%; 0.02%; 0.04%; 0.08%; 0.16%; 0.32%. Proteins were run on 8%–16% polyacrylamide gradient blue-native gels to visualize the free NDUFA4 protein subunit (left panel) and were also probed for MTCO1 to examine the complex IV associations (right panel). (C) Mitochondrial membrane proteins (10 μg), extracted using 0.08% DDM in order to verify colocalization of the NDUFA4 protein with complex IV (also see Figure 4B), were applied and run on 3%–12% polyacrylamide gradient blue-native gels using the muscle tissue of III-4 and a control. For two-dimensional analysis, a single lane was excised from the gel, denatured, and resolved by denaturing electrophoresis in the second dimension on an SDS polyacrylamide gel, prior to immunoblotting. One-dimensional native blots are shown above two-dimensional denatured blots to aid identification of protein spots. Blots were probed with specific antibodies against complex I (anti-NDUFA9), complex III (anti-UQCRC2), complex IV (anti-MTCO1, anti-MTCO2), and NDUFA4. Native markers were obtained by reprobing one-dimensional blots for SDHA to detect complex II (∼125 kDa) and ATP5A1 to detect holocomplex V (∼600 kDa), the F_1_ portion of complex V (∼400 kDa), and the free subunit (55 kDa). CI, complex I; CIII_2_, complex III dimer; CIV, complex IV; *a*, MTCO1; *b*, MTCO2; *c*, NDUFA4; *d*, UQCRC2; *e*, NDUFA9.

In-gel activity staining of blue-native gels supported spectrophotometric analysis of respiratory chain enzymes with normal complex I (NADH:ubiquinone oxidoreductase) and reduced complex IV in the muscle tissue of affected subjects (III-4 and III-6, Figure 4A). Recent work suggests an interaction of NDUFA4 with complex IV that is disrupted when high concentrations (>1.5%) of n-dodecyl β-D-maltoside (DDM), the standard detergent used to purify complex IV for crystallization purposes, are used (Balsa et al., 2012). To establish the optimal concentration of DDM required to analyze NDUFA4 as part of the COX holoenzyme, we performed western blot analysis of blue-native polyacrylamide gels loaded with 10 μg of mitochondrial enriched protein pellets extracted from control muscle tissue using serial dilutions of DDM for mitochondrial membrane protein extraction (Schägger, 1995; Williams et al., 2004). Blots were developed with antibodies directed against NDUFA4 and MTCO1. This analysis demonstrated that, in our hands, dissociation of NDUFA4 subunit from complex IV occurred when >0.08% DDM was used to extract mitochondrial membrane proteins, whereas ≤0.08% DDM allowed detection of NDUFA4 as part of complex IV (Figure 4B). We subsequently proceeded with western blot analysis of respiratory chain enzymes extracted using 0.08% DDM in order to verify interaction of the NDUFA4 protein with complex IV (Figure 4C). Analysis of one-dimensional blots of blue-native gels showed complex IV holoenzyme was present in the muscle tissue of III-4 with no abnormal subassemblies, whereas one- and two-dimensional blots confirmed association of NDUFA4 with complex IV in the control. NDUFA9, a known structural accessory subunit of complex I, migrated separately to NDUFA4 on the two-dimensional blots and appeared as a double spot in the patient sample (Figure 4C). Further experiments with human cell cultures (data not shown) indicated that the faster migrating spot represents a proteolytic truncation of NDUFA9, possibly resulting from prolonged storage of the patient’s muscle sample.

## Discussion

We studied a family with COX-deficient Leigh syndrome not explained by any previously described gene mutation. Through a combined homozygosity mapping and whole-exome sequencing approach, we identified a homozygous splice donor site mutation in *NDUFA4*, a gene previously considered to encode a subunit of respiratory chain complex I. Transcriptional studies demonstrated the c.42+1G → C transversion significantly reduces wild-type *NDUFA4* mRNA and produces an aberrant transcript that predicts a translated protein comprising 14 in-frame amino acids followed by 35 out-of-frame amino acids. Western blot analysis of denaturing gels and immunocytochemistry revealed undetectable steady-state NDUFA4 protein levels, indicating that this mutation causes a loss-of-function effect in the homozygous state.

The existence of some very-low-level wild-type *NDUFA4* transcripts in patients harboring the c.42+1G → C mutation might explain the slower natural history and the comparatively milder clinical phenotype observed; most previously reported patients with nuclear-encoded COX deficiency exhibit severe phenotypes resulting in early childhood death. Interestingly, mutant transcript alone was detected in cultured skin fibroblasts, despite apparently normal fibroblast COX activity observed in III-4 using a glucose-containing culture medium. Cells cultured with galactose as a carbon source are not able to produce ATP through glycolysis and are entirely dependent on oxidative phosphorylation (OXPHOS) for their ATP synthesis (Petrova-Benedict et al., 1992). We therefore repeated the assays with cells grown in galactose-containing medium for 6 days in order to accentuate the underlying defect in OXPHOS. In contrast to III-4 fibroblasts grown in glucose-containing medium, III-4 fibroblasts grown in galactose-containing medium showed a clear COX deficiency. These observations indicate that current diagnostic procedures that typically use glucose as a carbon source in the culture medium may not detect a reduction in COX activity.

Balsa and colleagues (2012) found that >1.5% of DDM disrupted the binding of NDUFA4 with complex IV in HeLa cell mitochondria, whereas in our hands >0.08% of DDM disrupted the binding in human muscle mitochondrial preparations (Figure 4B). In titration experiments with HeLa cells, we found that >0.04% of DDM disrupted the binding (data not shown). The discrepancy in the concentration of DDM required to dissociate NDUFA4 from the COX enzyme complex may be explained by differences in the buffer conditions used. We carried out our DDM extractions in 1 M ε-amino-n-caproic acid, 50 mM bistris (pH 7.0) (Schägger, 1995). The buffer conditions used by Balsa and colleagues were not specified.

Our data confirm that NDUFA4 is not required for assembly of the other COX subunits because we detected the fully assembled COX enzyme complex with no abnormal subassemblies on native gels of patient mitochondrial membrane proteins. In addition, the observation that COX activity is retained following its purification (Sinjorgo et al., 1987) using concentrations of DDM now known to disrupt NDUFA4 implies that NDUFA4 is not directly required for catalysis per se. However, deficiency of NDUFA4 clearly impairs COX activity in the muscle tissue and cultured skin fibroblasts in our patients harboring homozygous loss-of-function *NDUFA4* mutations. We suggest NDUFA4 is a subunit of COX rather than an assembly factor because blots of two-dimensional blue-native polyacrylamide gels demonstrated a clear association between NDUFA4 and the COX enzyme complex. We also propose that NDUFA4 is required during the biogenesis of COX to become an active enzyme and remains loosely associated with the enzyme complex. Apparently, a critical interaction occurs between NDUFA4 and the remaining COX subunits during biogenesis of the enzyme complex that is essential for COX activity. For example, it is possible that NDUFA4 facilitates folding of MTCO1 or is involved in addition or modification of prosthetic groups that affects downstream catalysis but not assembly of the enzyme. The precise nature of this interaction requires further study.

The discovery that *NDUFA4* mutations cause human COX deficiency indicates an important role of the NDUFA4 protein in COX function. Our findings are supported by recent mouse and human cell line evidence that the NDUFA4 protein is actually an additional COX subunit (Balsa et al., 2012), despite its previously accepted role as an accessory subunit of complex I (Carroll et al., 2002). This has important implications for understanding the precise biological function of the NDUFA4 protein and its interaction with the other COX polypeptide subunits and assembly factors. We suggest that analysis of the *NDUFA4* gene should be considered in all patients with unexplained COX deficiency.

## Experimental Procedures

### Subjects

The proband (III-4, Figure 1A; Tables 2 and 3) was the fourth child of healthy first-cousin Pakistani parents. He presented with lactic acidosis at birth following a normal pregnancy and full-term delivery. Birth weight was 2.7 kg. There was initial normal motor development, but gait was later considered “stiff,” and at 24 months he was diagnosed with a spastic diplegia. Speech was delayed and there were moderate learning difficulties. Aged 13 years, he suffered his first generalized tonic-clonic seizure and subsequently developed treatment-resistant myoclonus. An electroencephalogram indicated a right temporal focus with interictal discharges over the right anterior temporal and frontocentral regions. At 17 years, plasma lactate was 2.4 mmol/l (reference <2.0), and cerebrospinal fluid (CSF) lactate was 3.2 mmol/l (reference <2.0). CSF protein was also elevated at 0.54 g/l (reference 0.1–0.3), as was CSF alanine at 45 μg/l (reference 16–36). Brain CT (13 years) and MRI (17 years) were both normal. However, a brain MRI at age 25 years demonstrated T2 hyperintensities in the deep white matter. Nerve conduction studies indicated there was a sensory axonal peripheral neuropathy. Muscle biopsy at age 17 years showed nonspecific changes, including increased fiber size variation and intense peripheral staining of some fibers on modified Gomori trichrome, although no true ragged red fibers. COX histochemistry was normal. Electron microscopy revealed lipid droplets in some fibers. Spectrophotometric assay of muscle confirmed significant COX deficiency (COX/citrate synthase [CS] ratio 0.008; controls 0.014–0.034), whereas enzyme assays of mitochondrial respiratory chain complex I and complex II+III (succinate: cytochrome *c* reductase), corrected for CS, were normal. Although fibroblast COX activity was initially found to be normal (COX/CS ratio 2.2; controls >1), a repeat assay using whole-cell lysate of cultured skin fibroblasts grown on galactose rather than glucose medium did demonstrate reduced COX activity (1.07 k/min/mg; control mean ± SD [n = 5]: 3.60 ± 0.24 k/min/mg). Complex I activity in fibroblasts grown under the same conditions was normal (37.3 nmol/min/mg; control mean ± SD [n = 5]: 19.4 ± 7.2 nmol/min/mg). Pyruvate dehydrogenase activity was retained in cultured skin fibroblasts, and mutations in *SURF1* (encoding the surfeit locus protein 1, MIM 185620), *POLG* (encoding the catalytic subunit of mitochondrial DNA polymerase gamma, MIM 174763), *C10orf2* (encoding the DNA helicase Twinkle, MIM 606075), and the entire mitochondrial genome were excluded. Most recent clinical examination at age 32 years showed short stature (5 ft 4 in) and a spastic gait. Cranial nerves were normal. Upper and lower limb examination revealed dystonic posturing and increased tone with pyramidal weakness (Medical Research Council [MRC] grade 4/5). Tendon reflexes were pathologically brisk in arms and legs and plantar responses extensor.

Three additional affected family members (III-3, III-6, and III-13, Figure 1A; Tables 2 and 3; Document S1) presented similarly at birth with congenital lactic acidosis. Two had delayed motor and language skills and developed a combination of dystonia, ataxia, and pyramidal tract signs in later life. All had mild-moderate learning difficulties. The brain MRI of all three patients was characteristic of Leigh syndrome with T2 hyperintensities present to variable degrees in the deep white matter, basal ganglia, thalami, and brainstem (Figure 1B, III-3 shown). Reduced COX staining in all muscle fibers was seen in III-13, and isolated COX deficiency was confirmed by spectrophotometric assay of the muscle tissue from III-6 (0.004 COX/CS; controls 0.014–0.034), with normal complex I and II+III activity corrected for CS. One individual (III-6) died of respiratory failure at age 26 years due to presumed extension of existing and/or new brainstem lesions, whereas a second (III-13) died at age 8 years 9 months following progressive neurological regression with a gradual loss of motor, language, and cognitive skills.

Standard protocol approvals, registration, and patient consents were obtained. The study was approved and performed under the ethical guidelines issued by our institutions. In addition to the index family, a further 20 COX-deficient probands were screened for mutations in *NDUFA4*.

### Histopathology

Muscle biopsies were performed following informed consent, snap-frozen at the bedside, and stored at −80°C until use. Standard histological and histochemical stains were used on cryostat sections as previously described (Rahman et al., 2000).

### Cell Culture

Skin biopsies were performed following informed consent. Human fibroblasts were established from skin explants and cultured routinely in Dulbecco’s modified Eagle’s medium, 4.5 g/l d-(+)-glucose, Glutamax-I (Life Technologies) supplemented with 10% fetal bovine serum, 110 μg/ml of sodium pyruvate, 50 U/ml of penicillin, 50 μg/ml of streptomycin, and 50 μg/ml of uridine. For biochemical assays and immunocytochemistry, the glucose in the medium was replaced with 4.5g/l of d-(+)-galactose for 6 days. Medium was changed every 4 days. Cells were grown routinely in plastic dishes at 37°C in a humidified atmosphere of 5% CO_2_ in air but cultured onto glass coverslips for 3 days prior to immunocytochemical investigations (Taanman et al., 2003). Cultures were checked for mycoplasma infection by staining prior to all experiments.

### Preparation of Crude Mitochondrial Fractions

Frozen muscle tissue was ground in homogenization buffer (320 mM sucrose, 1 mM EDTA, 10 mM Tris-HCl [pH 7.4]) in a glass homogenizer manually. Muscle homogenates were centrifuged at 600 × *g* for 10 min at 4°C. The supernatants were stored on ice and the pellets were resuspended in homogenizing buffer, homogenized, and centrifuged at 600 × *g* for 10 min at 4°C. Both the supernatants were mixed and centrifuged at 12,000 × *g* for 10 min at 4°C. Crude mitochondrial fractions were isolated from ∼10 × 10^6^ cells of the fibroblast cultures by differential centrifugation (Rickwood et al., 1987). The mitochondrial enriched protein pellets were subsequently used for all biochemical assays (apart from spectrophotometric enzyme assay of cultured skin fibroblasts grown on galactose medium where whole-cell lysates were used), in-gel activity staining, and western blot analysis of SDS-denaturing and blue-native polyacrylamide gels. Protein concentrations were determined by Bradford assay.

### Biochemical Assays

Spectrophotometric enzyme assays of mitochondrial respiratory chain complex I, complex II+III, and complex IV activities corrected for CS were performed as previously described (Hargreaves et al., 2002). In-gel activity of complex I and IV was measured as previously described (Zerbetto et al., 1997). The staining intensity of each band was quantified using AlphaEase FC software from Alpha Innotech.

### Genetics Investigations

#### Homozygosity Mapping

Total genomic DNA (gDNA) was extracted from peripheral blood leucocytes using standard extraction protocols. We genotyped subjects II-2, III-1, III-2, III-3, III-4, and III-6 using the Illumina 740K OmniExpress single nucleotide polymorphism (SNP) array according to the manufacturer’s instructions and our previous publications (Paisan-Ruiz et al., 2009). All files were visually examined in GenomeViewer tool within BeadStudio v3.1 Genotyping module (Illumina), where two metrics were assessed: log *R* ratio and B allele frequency. The log *R* ratio gives an indirect measure of copy number of each SNP by plotting the ratio of observed to expected hybridization intensity. B allele frequency plots the proportion of times an allele is called B at each SNP locus: thus, the expected values are 1.0 (B/B), 0.5 (A/B), and 0.0 (A/A). These two statistics allow visualization of copy number changes and homozygosity mapping. After quality control, our first analysis was visual inspection to determine whether any large insertions/deletions were present, and whether any extended regions of homozygosity were shared by affected individuals and absent in unaffected family members. Subsequently, more detailed analysis used PennCNV to collate the copy number variant (CNV) data into Excel tables and to examine the CNV data to look for unique smaller insertions/deletions. The genotyping data with AA, AB, and BB were then saved as excel files for affected and unaffected individuals so that shared regions could be easily analyzed.

#### Whole-Exome Sequencing and Bioinformatic Analyses

Whole-exome sequencing was performed within the UK10K project. DNA (1–3 μg) was sheared to 100–400 bp using a Covaris E210 or LE220 (Covaris). Sheared DNA was subjected to Illumina paired-end DNA library preparation and enriched for target sequences (Agilent Technologies; Human All Exon 50 Mb - ELID S02972011) according to manufacturer’s recommendations (Agilent; SureSelectXT Automated Target Enrichment for Illumina Paired-End Multiplexed Sequencing). Enriched libraries were sequenced using the HiSeq platform (Illumina) as paired-end 75 base reads according to manufacturer’s protocol. The mean coverage of the exomes was 74 times. The Burrows-Wheeler Aligner (Li and Durbin, 2009) was used for alignment to the human reference genome build UCSC hg19 (Grch37). To improve raw alignment BAMs for SNP calling, we realigned around known (1000 Genomes pilot) indels and recalibrated base quality scores using GATK (DePristo et al., 2011). Base alignment quality tags were added using SAMtools calmd. BAMs for each sample were merged and duplicates marked using Picard. Variants (SNPs and indels) were called on each sample individually with both SAMtools mpileup (0.1.17) (Li et al., 2009) and GATK UnifedGenotyper (1.3.21) (McKenna et al., 2010), restricted to exon bait regions plus or minus a 100 bp window. Various quality filters were applied to each of the callsets separately. Calls were then merged, giving preference to GATK information when possible. Calls were annotated with 1000 Genomes allele frequencies, dbSNP132 rsids, and earliest appearance in dbSNP. Functional annotation was added using Ensembl Variant Effect Predictor v2.2 against Ensembl 64 and included coding consequence predictions, SIFT, PolyPhen and Condel annotations, and GERP and Grantham Matrix scores. Variant filtering of the single-nucleotide variants and indels was done as previously described on the basis of autosomal recessive inheritance. The filtering of the variants was done very stringently, excluding variants in dbSNP132, 1000 Genomes, synonymous, and other noncoding variants apart from essential splice site changes. Variants were also excluded if present in either the remaining UK10K rare disease cohort (823 exomes at the time of the analysis) or the NHLBI ESP database.

#### Sanger Sequencing

Confirmation and segregation of the c.42+1G → C mutation and screening of 20 additional COX-deficient probands for mutations in *NDUFA4* was assessed using standard PCR-based sequencing. Amplitaq Gold 360 Mastermix (Applied Biosystems) was used for all PCRs. Oligonucleotides for *NDUFA4* PCR amplification and sequence analysis in gDNA are listed in Table S4. M13 sequences to prime sequencing reactions were added to the 5′ end of the primers. Amplification of *NDUFA4* fragments for sequencing of cDNA was performed using a forward primer annealing in exon 1 (5′-GTCAGGCCAAGAAGCATCC-3′) and a reverse primer annealing in exon 4 (3′-TTCAGCTTGCTGTAATCCACA-5′). Primers were designed using Primer3 (http://frodo.wi.mit.edu/primer3/), and Sanger sequencing was performed using M13 primers and BigDye Terminator v.1.1 cycle sequencing kit (Applied Biosystems). The samples were run on a 3730xl DNA Analyzer, assembled, and analyzed using Seqscape v2.5 software (Applied Biosystems).

#### Transcriptional Analysis

RNA was extracted from whole blood using the QIAGEN/PreAnalytix blood RNA system and from cultured cells using the QIAGEN/RNeasy Mini Kit following the manufacturer’s instructions. cDNA was synthesized from ∼1 μg RNA in a 20 μl reaction using the Applied Biosystems high-capacity cDNA reverse transcription kit, following the manufacturer’s instructions.

#### Polyacrylamide Gel Electrophoresis of cDNA

Amplified cDNA fragments were resolved on a denaturing 7% polyacrylamide gel and stained with silver as previously described (Sambrook and Russell, 2001).

### Protein Analysis

#### Immunocytochemistry

Immunocytochemistry of the NDUFA4/MTCO1 and NDUFA4/MitoTracker Red CM-H_2_XRos- (Life Technologies) stained cells was carried out as described (Taanman et al., 1997b), except that anti-MTCO1 (clone 1D6E11A8, Abcam) was detected with goat anti-mouse IgG Alexa Fluor 488 (Life Technologies), anti-NDUFA4 (Stratech Scientific Ltd, AssaybioTech cat. no. C16821) with goat anti rabbit-IgG Alexa Fluor 594 or 488 (Life Technologies), and nuclei were counterstained with 1 μg of 4′,6-diamidino-2-phenylindole (Sigma) per ml of Citifluor-glycerol-PBS solution (Agar Scientific).

#### Western Blot Analysis of Denaturing and Blue-Native Polyacrylamide Gels

We evaluated protein steady-state levels using western blot analysis of SDS-denaturing polyacrylamide gels probed with antibodies directed against NDUFA4 and MTCO2 (clone 12C4F12, Abcam), performed as previously described (Williams et al., 2004).

To establish the optimal concentration of DDM required to analyze NDUFA4 as part of COX holoenzyme, we performed western blot analysis of 8%–16% polyacrylamide gradient blue-native gels loaded with 10 μg of mitochondrial protein extracted from control muscle tissue using serial dilutions of DDM (0.01%, 0.02%, 0.04%, 0.08%, 0.16%, and 0.32%) for mitochondrial membrane protein extraction (Schägger, 1995; Williams et al., 2004). Blots were developed with antibodies directed against NDUFA4 and MTCO1. Mitochondrial membrane proteins (10 μg), extracted using 0.08% DDM in order to verify colocalization of the NDUFA4 protein with complex IV, were subsequently applied and run on 3%–12% polyacrylamide gradient blue-native gels. For two-dimensional analysis, a single lane was excised from the gel, denatured with 1% SDS and 2% β-mecaptoethanol, and resolved by denaturing electrophoresis in the second dimension on an SDS polyacrylamide gel, prior to immunoblotting. Blots were probed with specific antibodies against complex I (anti-NDUFA9; clone 20C11B11B11, Abcam), complex III (anti-UQCRC2; clone 13G12AF12BB11, Abcam), complex IV (anti-MTCO1, anti-MTCO2), and NDUFA4. Native markers were obtained by reprobing one-dimensional blots for SDHA (anti-SDHA clone 2E3GC1FB2AE2, Abcam) to detect complex II (∼125 kDa) and ATP5A1 (anti-ATP5A1 clone 7H10BD4F9, Abcam) to detect holocomplex V (∼600 kDa), the F_1_ portion of complex V (∼400 kDa) and the free subunit (55 kDa).

## Figures and Tables

**Table 1:** Identification of Shared Candidate Genes for Cytochrome *c* Oxidase Deficiency in Subjects III-4 and III-6 with Exome Resequencing

Filter	III-4	III-6	Shared Variants
Total number of SNVs and indels	140,456	140,859	78,004
Homozygous SNVs and indels	50,868	50,832	28,320
Number of homozygous PASS SNVs and indels	26,379	25,289	19,389
Novel homozygous PASS SNVs and indels	442	412	190
Functional novel homozygous PASS SNVs and indels	46	40	13
Functional novel homozygous PASS SNVs and indels with predicted mitochondrial localization	2	2	1
	*IARS*	*FTSJ2*	
	*NDUFA4*	*NDUFA4*	*NDUFA4*

Indels, insertions/deletions; SNVs, single nucleotide variants. Novel, not reported to dbSNP132 and/or 1000 Genomes, the remaining UK10K rare disease cohort (823 exomes at the time of the analysis), or the NHLBI Exome Sequencing Project database. Functional, nonsynonymous coding and/or loss-of-function variants.

See also Tables S2 and S3.

**Table 2:** Clinical Characteristics of Subjects III-3, III-4, III-6, and III-13 with Homozygous c.42+1G → C Splice Donor Site Mutations in *NDUFA4*

								Clinical Manifestations		
Subject	Age (years)	Clinical Phenotype	DD	FTT	LD	Dystonia	Myoclonus	Ataxia	UMN Signs	Seizures
III-3	32	CLA, LS	L	+	+	+	−	−	−	−
III-4	34	CLA, LS	L, M	−	+	+	+	+	+	+
III-6	Died 26	CLA, LS	L	−	+	−	−	−	−	−
III-13	Died 8	CLA, LS	L, M	+	+	+	−	+	+	−

CLA, congenital lactic acidosis; DD, developmental delay; FTT, failure to thrive; L, language; LD, learning difficulties; LS, Leigh syndrome; M, motor; UMN, upper motor neuron. +, present; −, absent.

**Table 3:** Laboratory, Neurophysiological, Imaging, Histopathological, and Biochemical Characteristics of Subjects III-3, III-4, III-6, and III-13 with Homozygous c.42+1G → C Splice Donor Site Mutations in *NDUFA4*

							COX/CS		COX (k/min/mg)
Subject	Plasma Lactate	CSF Lactate	NCS	Brain MRI	Brain MRS	Muscle Pathology	Muscle	Fibroblasts (glucose)	Fibroblasts (galactose)
III-3	3.5–6.6	—	sensory axonal neuropathy	aged 32: ↑T2 signal deep WM, thalami, brainstem, cerebellum	—	—	—	—	—
III-4	1.6–2.4	3.2	sensory axonal neuropathy	aged 17: normal Aged 25: ↑T2 signal deep WM	—	↑lipid	0.008	2.2	1.07
III-6	2.5–4.7	—	—	aged 25: ↑T2 signal parietal WM, BG, thalami, brainstem	—	↑lipid	0.004	—	—
III-13	2.1–8.5	6.4	—	aged 2: ↑T2 signal parietal WM	↑lactate peak in BG	global ↓COX, ↑lipid	—	1.7	—

BG, basal ganglia; COX, cytochrome *c* oxidase; CS, citrate synthase; CSF, cerebrospinal fluid; NCS, nerve conduction studies; MRS, magnetic resonance spectroscopy; WM, white matter. Reference ranges: plasma lactate 0.7–2.1 mmol/l; CSF lactate <2 mmol/l; COX/CS in muscle 0.014–0.034; COX/CS in glucose-grown fibroblasts >1; COX/protein in galactose-grown fibroblasts 3.35–3.88 k/min/mg. —, test not performed.

## Supplemental Information

Table S1. Two Large Regions of Homozygosity Shared By Subjects III-3, III-4, and III-6, Related to Figure 1

Table S2. Coverage of the Linkage Loci Calculated from the BAM Files with coverageBed for Subject III-4, Related to Table 1 The coverageBed tool can be found at http://code.google.com/p/bedtools/.

Table S3. Coverage of the Linkage Loci Calculated from the BAM Files with coverageBed for Subject III-6, Related to Table 1 The coverageBed tool can be found at http://code.google.com/p/bedtools/.

Table S4. Oligonucleotides for *NDUFA4* PCR Amplification and Sequence Analysis in gDNA, Related to Figure 2

Document S1. Case Descriptions and UK10K Rare Group Authors and Their Affiliations
