# Supplementary material for: NDUFA4 Mutations Underlie Dysfunction of a Cytochrome c Oxidase Subunit Linked to Human Neurological Disease
Source: Cell Rep. 2013 Jun 27;3(6):1795–805. doi: 10.1016/j.celrep.2013.05.005 (PMC3701321; doi:10.1016/j.celrep.2013.05.005)
Supplement: Table S1. Two Large Regions of Homozygosity Shared By Subjects III-3, III-4, and III-6, Related to Figure 1 [file mmc1.docx]

**Table S1:** Two large regions of homozygosity shared by subjects III-3, III-4, and III-6

| **Region 1 (Ch 7p: 9,219,283-13,801,764)** | **Genes (15)** |
| --- | --- |
|  | *PER4* |
|  | ***NDUFA4*** |
|  | *PHF14* |
|  | *BC040327* |
|  | *THSD7A* |
|  | *KIAA0960* |
|  | *TMEM106B* |
|  | *VWDE* |
|  | *AK027618* |
|  | *AK075525* |
|  | *BC075797* |
|  | *CR592342* |
|  | *KIAA1905* |
|  | *SCIN* |
|  | *ARL4A* |
| **Region 2 (Ch 7p: 19,034,191-29,250,335)** | **Genes (92)** |
|  | *CR623750* |
|  | *TWIST1* |
|  | *BC043576* |
|  | *FERD3L* |
|  | *TWISTNB* |
|  | *TMEM196* |
|  | *ABCB5* |
|  | *SP8* |
|  | *ITGB8* |
|  | *MACC1* |
|  | *RPL23P8* |
|  | *SP4* |
|  | *DNAH11* |
|  | *CDCA7L* |
|  | *RAPGEF5* |
|  | *MGC87042* |
|  | *AK123961* |
|  | *LOC541472* |
|  | *IL6* |
|  | *C7orf30* |
|  | *TOMM7* |
|  | *SNORD93* |
|  | *KLHL7* |
|  | *NUPL2* |
|  | *AK057873* |
|  | *GPNMB* |
|  | *BC065766* |
|  | *IGF2BP3* |
|  | *IMP-3* |
|  | *TRA2A* |
|  | *hAWMS1* |
|  | *CLK2P* |
|  | *CCDC126* |
|  | *C7orf46* |
|  | *STK31* |
|  | *FAM126A* |
|  | *DRCTNNB1A* |
|  | *AK054880* |
|  | *RPS2P32* |
|  | *DKFZp434E052* |
|  | *DFNA5* |
|  | *NPY* |
|  | *MPP6* |
|  | *CYCS* |
|  | *ICERE-1* |
|  | *OSBPL3* |
|  | *C7orf31* |
|  | *NPVF* |
|  | *SNX10* |
|  | *MIR148A* |
|  | *NFE2L3* |
|  | *HNRNPA2B1* |
|  | *CBX3* |
|  | *hp1-gamma* |
|  | *SNX10* |
|  | *LOC441204* |
|  | *KIAA0087* |
|  | *C7orf71* |
|  | *LOC285941* |
|  | *SKAP2* |
|  | *HOXA2* |
|  | *HOXA3* |
|  | *AK311383* |
|  | *BC035889* |
|  | *HOXA5* |
|  | *HOXA6* |
|  | *AX747263* |
|  | *DQ655986* |
|  | *AK096334* |
|  | *HOXA7* |
|  | *HOXA9* |
|  | *MIR196B* |
|  | *HOXA10* |
|  | *HOXA11* |
|  | *HOXA11AS* |
|  | *HOXA13* |
|  | *AK093987* |
|  | *HIBADH* |
|  | *EVX1* |
|  | *TSL-A* |
|  | *TAX1BP1* |
|  | *BC034444* |
|  | *NS5ATP1* |
|  | *JAZF1* |
|  | *LOC402644* |
|  | *CREB5* |
|  | *BC087859* |
|  | *KIAA0644* |
|  | *DQ601810* |
|  | *CPVL* |
|  | *AK124888* |
|  | *CHN2* |
