# Supplementary material for: NDUFA4 Mutations Underlie Dysfunction of a Cytochrome c Oxidase Subunit Linked to Human Neurological Disease
Source: Cell Rep. 2013 Jun 27;3(6):1795–805. doi: 10.1016/j.celrep.2013.05.005 (PMC3701321; doi:10.1016/j.celrep.2013.05.005)
Supplement: Table S4. Oligonucleotides for NDUFA4 PCR Amplification and Sequence Analysis in gDNA, Related to Figure 2 [file mmc4.pdf]

Table S4. Oligonucleotides for *NDUFA4* PCR Amplification and Sequence Analysis in gDNA, Related to Figure 2

	Forward Primers (5’ to 3’)	Reverse Primers (3’ to 5’)
**Exon 1**	GACCAGGTCAGGACGAACAT	GTCTGACGGACGGTAAGTGG
**Exon 2**	ATCTCTCGTTGGCCAGTGTT	TTTACATGTGCCGAGAACCA
**Exon 3**	AAAGGTTTTGTGAAAGCCAGA	TCTCTGGGGATAAGGCTGAA
**Exon 4**	CATTGAAGGCTGGAATTTTAGG	TCCAGTTCCAGTTGTTATGTATGC
