# Supplementary material for: NDUFA4 Mutations Underlie Dysfunction of a Cytochrome c Oxidase Subunit Linked to Human Neurological Disease
Source: Cell Rep. 2013 Jun 27;3(6):1795–805. doi: 10.1016/j.celrep.2013.05.005 (PMC3701321; doi:10.1016/j.celrep.2013.05.005)
Supplement: Document S1. Case Descriptions and UK10K Rare Group Authors and Their Affiliations [file mmc5.pdf]

Supplemental Information

Subject III-3 was the third child of first cousin healthy Pakistani parents. She was born at term following a normal delivery. Birth weight was 2.3 kg. Growth was poor in the neonatal period but no underlying cause was identified. She had a systolic murmur and mild pulmonary stenosis was found on cardiac catheterization. At 6 months lactic acidosis was noted, with plasma lactate levels ranging from 3.5-6.6 mmol/L and elevated plasma alanine. There was also evidence of proximal renal tubular acidosis. This was treated with oral sodium bicarbonate. At 10 months she had mild motor delay and she was later diagnosed with speech delay and moderate learning difficulties. There is no history of seizures.

Neurophysiology showed evidence of a predominantly sensory axonal peripheral neuropathy mainly affecting lower limbs, with no evidence of myopathy. Brain MRI (Figure 1B) showed multiple scattered foci of T2 hyperintensities within the deep white matter of both cerebral hemispheres, medial thalami, at the level of the superior cerebellar peduncles, corticospinal tracts of the pons, right middle cerebellar peduncles, cerebellar hemispheres and descending tracts at the levels of the medulla oblongata. She has not had a muscle biopsy.

Most recent clinical examination aged 34 years showed limited speech and understanding. There was mild upper limb and gait dystonia, but neurological assessment was otherwise normal.

Subject III-6 was the sixth child (second dizygotic twin) of first cousin Pakistani parents. She presented at birth, following an uneventful pregnancy, with lactic acidosis which resolved with sodium bicarbonate. Birth weight was 1.9 kg. She was discharged one month later and continued on oral sodium bicarbonate until aged 7 years. She was subsequently diagnosed with mild learning difficulties, but was otherwise medically stable until the age of 25 years when she developed hypertension and suffered a respiratory arrest requiring tracheal intubation and admission to intensive care. Brain magnetic resonance imaging (MRI) demonstrated T2 hyperintensities in the anterior lateral medulla, posterior pons, and the thalami, parietal white matter and basal ganglia. Muscle biopsy demonstrated a mild increase

in lipid but was otherwise normal. Spectrophotometric assay of muscle confirmed cytochrome-*c* oxidase (COX) deficiency (COX/citrate synthase [CS] ratio 0.004; controls 0.014-0.034). She was eventually extubated and established on nocturnal non-invasive ventilation. She required a protracted period of rehabilitation and was discharged 8 months following her initial admission when she was alert and conversing, although was dependent for most care needs. Unfortunately, she later died aged 26 years of a second respiratory arrest caused by presumed extension of her existing or new brainstem lesions.

Subject III-13 was the seventh child of healthy Pakistani parents who are second cousins. His six siblings are all well. He was a second dizygotic twin and was born by forceps delivery for breech presentation at 37 weeks' gestation. At birth he was floppy and bradycardic and required cardiopulmonary resuscitation. Apgar scores were 3 at 1 minute and 9 at 5 minutes. He had a mixed respiratory and metabolic acidosis (pH 6.99, pCO2 8.74, bicarbonate 14 mmol/l, base deficit 14.8 mmol/l) and was admitted briefly to the neonatal unit for headbox oxygen treatment, but his respiratory symptoms had settled by 24 hours of age. At 4 months poor growth and mild motor developmental delay were noted. At 2 years he was referred to the metabolic unit because of poor growth, developmental delay and lactic acidosis. At 3 years he developed dystonic posturing of the left arm and respiratory distress due to lactic acidosis, which responded to sodium bicarbonate (1 mmol/kg/day). He had frequent upper respiratory infections during the first 5 years of life. Neurological regression occurred from 4 years, with gradual but progressive loss of skills (walking, speech, cognition) over the next four years until his death at 8 years and 9 months of age. Examination at 4 years revealed bilateral horizonatal nystagmus, optic atrophy, gait ataxia, tremor, thin musculature and brisk tendon reflexes. At 8 years he also had a four-limb dystonia. He never had seizures or myoclonus.

Plasma lactate ranged from 2.1-8.5 mmol/L (reference range 0.7-2.1 mmol/L) and CSF lactate was elevated at 6.4 mmol/L (normal <2 mmol/L) with a paired blood lactate of 5.8 mmol/L. Echocardiography was normal, and there was no evidence of renal tubulopathy or hearing impairment. MRI brain at 2 years demonstrated abnormal signal in the parietal white matter with no convincing involvement of the basal ganglia or brainstem, whilst magnetic resonance spectroscopy showed an

increased lactate peak in the basal ganglia. Muscle histology at 2 years revealed reduced histochemical staining of COX in all muscle fibres and increased lipid in type I fibres. Respiratory chain enzyme activities were not formally determined in muscle, but fibroblast COX activity was normal at 43 nmole/mg protein/min (reference range 30-90) with a normal COX/citrate synthase ratio of 1.7 (normal >1).

UK10K Rare Group Authors and Affiliations

Al-Turki, Saeed[1, 2]

Anderson, Carl[1]

Antony, Dinu[3]

Barroso, Inês[1]

Beales, Phil[3]

Bentham, Jamie[4]

Bhattacharya, Shoumo[4]

Carss, Keren[1]

Chatterjee, Krishna[5]

Cirak, Sebahattin[6]

Cosgrove, Catherine[4]

Danecek, Petr[1]

Durbin, Richard[1]

Fitzpatrick, David[7]

Floyd, Jamie[1]

Foley, A. Reghan[6]

Franklin, Chris[1]

Futema, Marta[8]

Humphries, Steve E.[8]

Hurles, Matt[1]

Joyce, Chris[1]

McCarthy, Shane[1]

Mitchison, Hannah M.[3]

Muddyman, Dawn[1]

Muntoni, Francesco[6]

O'Rahilly, Stephen [5]

Onoufriadis, Alexandros[3]

Payne, Felicity[1]

Plagnol, Vincent[9]

Raymond, Lucy[10]

Savage, David B.[5]

Scambler, Peter[3]

Schmidts, Miriam[3]

Schoenmakers, Nadia[5]

Semple, Robert[5]

Serra, Eva[1]

Stalker, Jim[1]

van Kogelenberg, Margriet[1]

Vijayarangakannan, Parthiban[1]

Walter, Klaudia[1]

Whittall, Ros[8]

Williamson, Kathy[7]

[1]The Wellcome Trust Sanger Institute, Wellcome Trust Genome Campus, Hinxton CB10 1HH, Cambridge, UK.

[2]Department of Pathology, King Abdulaziz Medical City, Riyadh, Saudi Arabia.

[3]Molecular Medicine Unit, UCL Institute of Child Health, London, WC1N 1EH, UK.

[4]Department of Cardiovascular Medicine and Wellcome Trust Centre for Human Genetics, Roosevelt Drive, Oxford, OX3 7BN, UK.

[5]University of Cambridge Metabolic Research Laboratories, and NIHR Cambridge Biomedical Research Centre, Institute of Metabolic Science, Addenbrooke's Hospital, Cambridge, CB2 0QQ, UK.

[6]Dubowitz Neuromuscular Centre, UCL Institute of child health & Great Ormond street Hospital, London, WC1N 3JH, UK.

[7]MRC Human Genetics Unit, MRC Institute of Genetic and Molecular Medicine, at the University of Edinburgh, Western General Hospital, Edinburgh, EH4 2XU, UK.

[8]Cardiovascular Genetics, BHF Laboratories, Rayne Building, Institute Cardiovascular Sciences, University College London, London WC1E 6JJ, UK.

[9]University College London (UCL) Genetics Institute (UGI) Gower Street, London, WC1E 6BT, UK.

[10]Dept. of Medical Genetics, Cambridge Institute for Medical Research, University of Cambridge, CB2 2XY, UK.

[11]Center for Research, Prevention and Treatment of Atherosclerosis, Department of Medicine, Hadassah Hebrew University Medical Center, Jerusalem, Israel.

[12]Department of Biomedical, Metabolic and Neural Sciences, University of Modena and Reggio Emilia, Via campi 287, I-41125 Modena/Italy.

[13]Department of Cardiology, Imperial College Health Services, Charing Cross Hospital, London.
